# Preparation of Elastic Macroporous Graphene Aerogel Based on Pickering Emulsion Method and Combination with ETPU for High Performance Piezoresistive Sensors

**DOI:** 10.3390/mi14101904

**Published:** 2023-10-05

**Authors:** Wei Zhao, Hao Chen, Yuqi Wang, Qing Zhuo, Yaopeng Liu, Yuanyuan Li, Hangyu Dong, Shidong Li, Linli Tan, Jianfeng Tan, Zhuo Liu, Yingru Li

**Affiliations:** 1 Key Laboratory of Green Manufacturing of Super-Light Elastomer Materials of State Ethnic Affairs Commission, Hubei Minzu University, Enshi 445000, China; zhaowei7719@outlook.com (W.Z.); yqwang1103@gmail.com (Y.W.); zhuoqing1w@outlook.com (Q.Z.); liyuanyuan5179@163.com (Y.L.); h.y.dong@outlook.com (H.D.); 1994008@hbmzu.edu.cn (S.L.); 2019042@hbmzu.edu.cn (L.T.); tanjf.china@outlook.com (J.T.); zhuoliu@hbmzu.edu.cn (Z.L.); 2 College of Intelligent Systems Science and Engineering, Hubei Minzu University, Enshi 445000, China; crt1193636304@outlook.com; 3 College of Chemical and Environmental Engineering, Hubei Minzu University, Enshi 445000, China; lyp4812@163.com

**Keywords:** Macroporous Graphene Aerogel, flexible sensors, piezoresistive materials, pickering emulsion, ETPU

## Abstract

High-performance pressure sensors provide the necessary conditions for smart shoe applications. In this paper, the elastic Macroporous Graphene Aerogel (MGA) was synthesized via the modified Hummers’ method, and it was further combined with Expanded-Thermoplastic polyurethane (ETPU) particles to assemble MGA-ETPU flexible sensors. The MGA-ETPU has a low apparent density (3.02 mg/cm^3^), high conductivity (0.024 S/cm) and fast response time (50 ms). The MGA-ETPU has a large linear sensing range (0–10 kPa) and consists of two linear regions: the low-pressure region (0 to 8 kPa) and the high-pressure region (8 to 10 kPa), with sensitivities of 0.08 kPa^−1^, and 0.246 kPa^−1^, respectively. Mechanical test results show that the MGA-ETPU sensor showed 19% reduction in maximum stress after 400 loading–unloading compression cycles at 40% strain. Electrical performance tests showed that the resistance of MGA-ETPU sensor decreased by 12.5% when subjected to sudden compression at 82% strain and returned to its original state within 0.05 s. Compared to existing flexible sensors, the MGA-ETPU sensors offer excellent performance and several distinct advantages, including ease of fabrication, high sensitivity, fast response time, and good flexibility. These remarkable features make them ideally suited as flexible pressure sensors for smart shoes.

## 1. Introduction

As technology advances and the demand for smart wearable devices grows, flexible sensors have become increasingly popular [1,2]. The functionality of smart shoes has evolved from simple pedometers to include features like gait recognition [3], environmental monitoring [4], and risk warning [5]. To accommodate these evolving functions, there is a growing demand for enhanced performance of flexible sensors [4,6]. Flexible sensors can be categorized into different types, such as resistive [7,8], capacitive [9,10], piezoelectric [11,12], and field-effect type [13,14]. Among these, piezoresistive flexible sensors due to their faster response speed and superior performance, making them widely used in various smart devices. Typically, piezoresistive flexible sensors consist of elastic matrix (which is mostly insulative) and conductive filler. However, it is important to note that some limitations exist with piezoresistive sensors. For instance, the proportion of conductive filler added affects the relationship between conductivity and flexibility of the flexible sensor. Specifically, adding too little conductive filler will result in poor conductivity and low sensitivity of the sensor. On the contrary, too much filler can make the polymeric composite become too hard, which is not favorable for comfortability. Yang et al. added different contents of carbon black (CB) to silicone rubber to improve the conductivity of the composite material. The result found that the conductivity and the hardness of the composite silicone rubber will increase with the increasing CB content [15]. Hydrogen bonds are formed between the silicon–oxygen bonds in the rubber molecular chain and the oxygen-containing functional groups on the surface of CB, which improves the hardness of the composite.

Various nanofillers have been employed in these sensors, including metal nanoparticles [16], metal nanowires [17], carbon nanotubes [18], and graphene. In recent years, research has shown that flexible sensors made of graphene using a three-dimensional structure have the advantages of high sensitivity, high conductivity, and easy signal acquisition are widely used in a variety of smart devices [19]. What is more, graphene can form three-dimensional elastic material independently, named as graphene aerogel, which is formed by stacking 2D graphene sheets, exhibiting different properties based on the preparation methods used [20]. For example, Ma et al. prepared MXene/rGO aerogel by mixing graphene oxide (GO) solution with MXene solution in different ratios and assembled MX/rGO flexible stress sensor by immobilizing the aerogel on flexible Polyimide (PI) electrodes, which has a high sensitivity, a fast response time, and a good stability, and can be used for detecting the subtle pulse beats of the wrist [21]. Wei et al. used a simple sol-gel-hydrothermal reduction method to prepare Polypyrrole/rGO films, which are more suitable for use in flexible wearable piezoresistive sensors, which have high sensitivity, short response time and short relaxation time, and can be used for detecting a variety of human pulse signals, as well as timbre differentiation of sounds [22]. Tan et al. plasma-treated cotton fabric to adsorb reduced graphene oxide (rGO) on the fabric surface, and prepared electrode circuits using laser cutting of polyimide (PI) covering the surface of cotton fibers, and assembled these two fibers to prepare flexible textile piezoresistive sensor (TPRSs), which have the advantages of high sensitivity, fast response time, and good stability [23]. Overall, graphene composite flexible sensors have many advantages such as high sensitivity, good mechanical properties, light weight, etc., and have great potential in the field of wearable piezoresistive flexible sensors, especially in smart shoes.

Here, we describe a flexible pressure sensor for smart shoe applications. In our previous work, we had developed Macroporous Graphene Aerogel (MGA) with good conductivity and very low density, which has the potential to be used as a flexible pressure sensor [20]. In addition, ETPU is a foamed material with high elasticity, high toughness, and low-density properties obtained by adding supercritical carbon dioxide to a reactor with thermoplastic polyurethane particles, which makes them an ideal material for the soles of smart shoes. We prepared a flexible pressure sensor for smart shoes by perfectly integrating MGA into ETPU.

## 2. Experimental Details

### 2.1. Materials

Natural graphite powders of 325 mesh were purchased from Qingdao Huatai Lubricant Sealing S&T Co., Ltd. (Qingdao, China). Potassium permanganate (KMnO_4_), 30 wt% hydrogen peroxide (H_2_O_2_), hydrochloric acid (HCl), 98 wt% sulfuric acid (H_2_SO_4_) and n-hexane were procured from Sinopharm Chemical Reagent Co., Ltd. (Shanghai, China).

### 2.2. Preparation of the Macroporous Graphene Aerogel

GO was utilized as the precursor, and synthesized via the modified Hummers’ method [24]. In detail, 70 mL of concentrated sulfuric acid was added to the beaker in an ice-water bath environment, followed by adding 7.5 g of potassium permanganate powder to the beaker in batches during continuous stirring, and after stirring, 2.5 g of graphite powder was added to the beaker and stirred well, and this process was needed to ensure that the suspension was at an environment of 5 °C. Subsequently, the reaction system was transferred to 35 ± 5 °C for low-temperature oxidation, and a dark brown viscous liquid was obtained after continuous stirring for 30 min. Then, the suspension was heated to 95 ± 5 °C while stirring for 15 min. To the reaction system, 420 mL of deionized water was rapidly added, and 15 mL of hydrogen peroxide was added in batches to remove excess potassium permanganate until the dispersion in the cup changed from dark brown to brownish yellow after no more bubbles were produced in the beaker. Then, the brown-yellow dispersion was filtered through a Brinell funnel to obtain a brown-yellow filter cake, which was then washed sequentially with 200 mL of dilute hydrochloric acid (1:10, *v*/*v*) to remove the metal ions, and then washed with 1000 mL of deionized water to remove the excess acid. The GO dispersion was obtained by the modified Hummers’ method described above.

**Figure 1:**
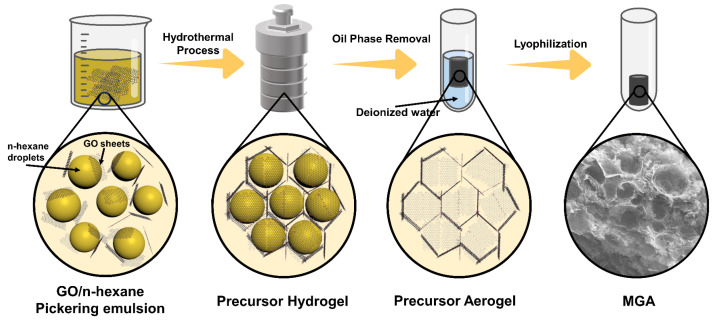
Preparation process of the Macroporous Graphene Aerogel (MGA).

After naturally dialyzing the GO dispersion for 1 week to remove impurity ions, the concentrated GO solution was obtained by centrifugation at 4000 rpm for 30 min to remove black precipitated impurities, followed by centrifugation at 10,000 rpm for another 30 min. To create a Pickering emulsion, 6 mL of the GO dispersion was mixed with 2 mL of n-hexane and stirred vigorously. The resulting emulsion was then placed in a hydrothermal reactor, which was subsequently maintained in an oven at 180 °C for 10 h. After cooling to room temperature, a hydrogel containing n-hexane drops was obtained. The hydrogel was immersed in deionized water and heated to 80 °C for 1 h to evaporate n-hexane in the hydrogel’s pores. The hydrogel was then subjected to low-temperature freezing treatment at −18 °C for 24 h. Finally, the hydrogel was freeze-dried in a vacuum freeze dryer. This freeze-drying process resulted in the formation of MGA, as shown in Figure 1.

### 2.3. Assemble of MGA-ETPU Sensor

**Figure 2:**
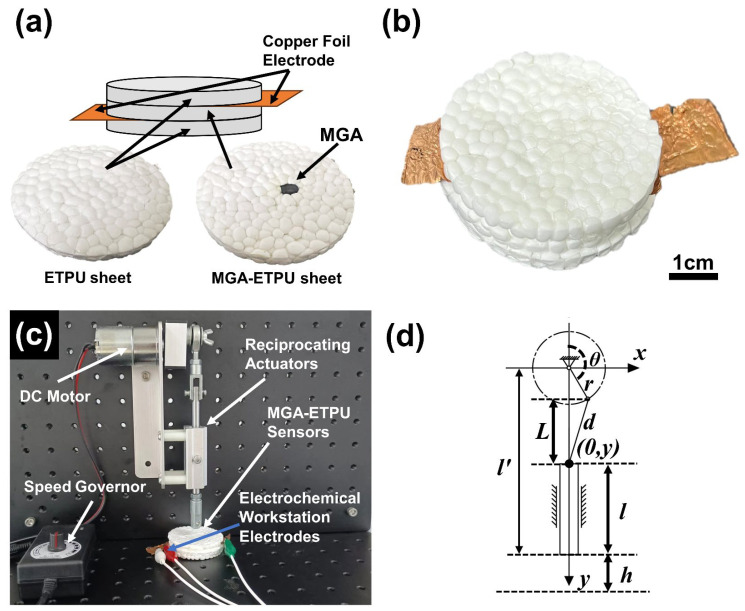
(**a**) Schematic structure of MGA-ETPU sensor; (**b**) Digital photo of MGA-ETPU sensor; (**c**) Reciprocating actuator unit connected to Electrochemical Workstation. (**d**) Mechanism motion diagram of reciprocating actuator.

To assemble MGA-ETPU, the copper foils were attached to the top and bottom sides of the MGA using conductive silver paste for enhancing the contact between the MGA and the copper foil, which is important for the sensor’s sensitivity (Figure 2a). MGA was placed in a circular mold (diameter 58 mm, thickness 5 mm) of a plate vulcanizer filled with Thermoplastic polyurethane foam beads (ETPU)particles. The temperature of the upper hot plate in the vulcanizer was set to 150 °C, while the temperature of the lower hot plate was set to 140 °C. The pressure applied during the hot-pressing process was 5 MPa, and the duration of the hot pressing was three minutes. After the hot-pressing process, the MGA and ETPU particles were bonded together, resulting in the formation of the MGA-ETPU sensor, as shown in Figure 2b. This sensor combines the properties of the MGA with the functionality of the TPU foam beads, making it suitable for flexible sensing applications. The MGA-ETPU sensor has an overall thickness of 14.74 mm and a mass of 6.449 g (without copper foil electrodes). In addition to this, a sample assembled with pure ETPU particles was fabricated as a control, which has an overall thickness of 15.06 mm and a mass of 6.227 g, and both samples have a diameter of 58 mm.

### 2.4. Characterization

The microscopic morphology of MGA was characterized by field emission scanning electron microscopy (Sirion-200, FEI, Hillsboro, Oregon, USA). The Raman spectra of MGA were recorded on a Laser confocal Raman Spectrometer (LabRam HR 800, Horiba Jobin Yvon, France, Paris) using a 532 nm laser set at a power density of 4.7 mW. X-ray photoelectron spectroscopy (XPS) analysis was performed using an instrument (ESCALAB 250XI, Thermo Fisher Scientific, Waltham, Massachusetts, USA). Thermogravimetric analysis (EXSTAR TG/DTA6300, SII, Japan, Tokyo) was used to study the thermal stability and components of MGA by heating the samples from 30 to 800 °C at a heating rate of 10 °C/min under 80 mL/min N_2_ atmosphere. The mechanical compressibility of the MGA-ETPU was carried out using a universal testing machine (TY800A, Tian Yuan Test Instrument, China, Yangzhou) with a compression rate of 5 mm/min. Samples of 58 mm (diameter) × 15 mm (thickness) were prepared for testing. Set the four-probe resistivity tester (BEST-300C, BEIGUANG, China, Beijing) to measure MGA resistivity and conductivity at a constant current source of 1 mA.

### 2.5. Piezoresistive Performance of MGA-ETPU Sensor

The MGA-ETPU sensor was placed on a dedicated rotary reciprocating device for reciprocating axial compression testing (Figure 2c). The current signal of the MGA-ETPU was acquired used an electrochemical workstation to evaluate the piezoresistive performance of the sensor to verify the sensitivity of the MGA-ETPU and its feasibility as a pressure sensor (CHI 760E, China, Shanghai).

The motion state of the connecting rod end of the rotary reciprocating device was analyzed, and the mechanism motion diagram was drawn and a Cartesian coordinate system was established with the center of the crank as the origin (Figure 2d), in which the crank length is *r* = 14.3 mm, the length of the connecting rod is *d* = 51 mm, and the coordinates of the end of the connecting rod are (0, *y_0_*) when the crank is moving to *θ* = 90°, then

(1) $$y_{0}=-\sqrt{d^{2}-r^{2}}$$

The coordinates of the end of the connecting rod are (0, *y*), and the longitudinal coordinates of the end of the connecting rod can be expressed as:

(2) $$y=rcos\theta -\sqrt{r^{2}{cos}^{2}\theta +y_{0}^{2}}$$

The value of the projection of the length of the connecting rod on the *y*-axis is *L*, the length of the compression rod is *l*, and the distance from the end of the compression rod to the bottom surface is *h*. When the crank connecting rod mechanism moves to the bottom limit position, the distance between the end of the compression rod and the bottom surface is *h*_0_ = 41.1 mm. At any moment, the value of the longitudinal coordinates of the end of the compression rod is:

(3) $$h=h_{0}-y_{0}+rcos(\omega t+\theta )-\sqrt{r^{2}{cos}^{2}(\omega t+\theta )+y_{0}^{2}}$$

The thickness of MGA-ETPU when it is not compressed is *H* = 12.3 mm, then the strain of MGA-ETPU can be expressed as:

(4) $$\varepsilon =\frac{h-H}{H}$$

Bring the Equation (3) into Equation (4), the strain expression of MGA-ETPU can be obtained as:

(5) $$\varepsilon =\frac{h_{0}-y_{0}-H+rcos(\omega t+\theta )-\sqrt{r^{2}{cos}^{2}(\omega t+\theta )+y_{0}^{2}}}{H}$$

## 3. Results and Discussion

### 3.1. Morphological and Structural Studies of MGA

**Figure 3:**
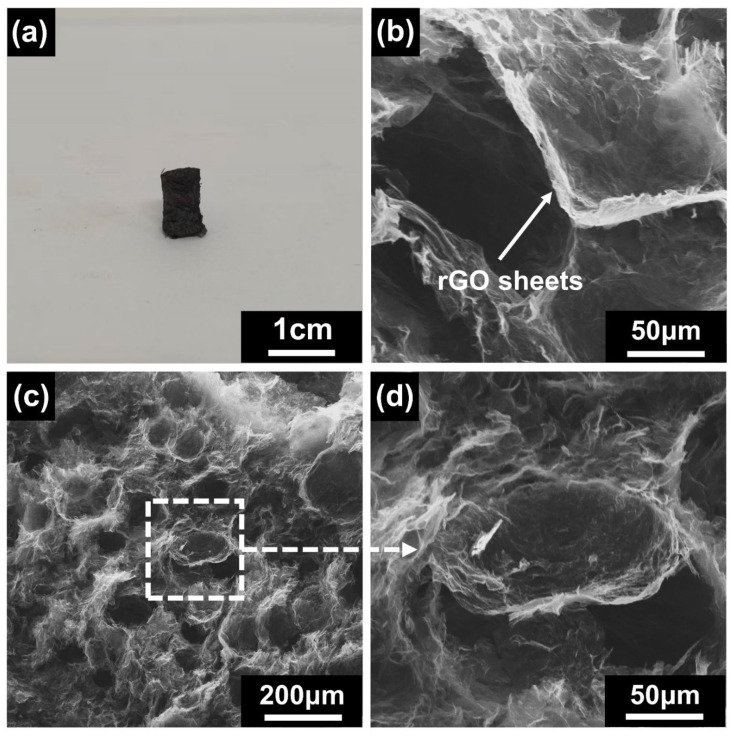
(**a**) MGA images; (**b**) rGO sheets form a three-dimensional network structure of MGA; (**c**,**d**) SEM images of MGA with different magnifications.

MGA obtained after the hydrothermal reduction process appeared as a black solid material (Figure 3a). This aerogel had an exceptionally low mass and density compared to other composite GO aerogels, with an apparent density of 3.02 mg/cm^3^. The extremely low density allowed for little mass variation in the ETPU sensors made by adding MGA, which could ensure the light weight, elasticity, and flexibility of smart ETPU products. 

The SEM images provide further insights into the microstructure of the MGA (Figure 3b–d). The presence of numerous irregular spherical closed pore structures can be observed in the images. These closed pores originated from the n-hexane oil droplets that were present in the Pickering emulsion. During the hydrothermal process, most of the n-hexane was confined within the Pickering emulsion system and served as a soft template within the emulsion system. The hydrothermal reduction treatment led to the reduction of graphene oxide (GO) sheets to reduced graphene oxide (rGO) sheets. Additionally, it caused micro-adjustments in the pore wall structure of the aerogel. As a result, the pore walls became folded, enabling them to withstand greater deformation (Figure 3b). The closed pore structures play a crucial role in maintaining the shape of the n-hexane oil droplets within the Pickering emulsion. The diameter of these closed pores was approximately 100 μm. This indicates that the rGO sheets and aggregates formed a three-dimensional network structure around the n-hexane oil droplets. The pore walls of this unique three-dimensional network structure were formed by the splicing of multilayer GO sheets. The structure can be likened to that of a polymer foam but with a wrinkled rGO film as the pore wall. This wrinkled structure is believed to contribute to the excellent elasticity exhibited by the final product. Overall, the SEM images illustrate the intricate and highly porous microstructure of the MGA, resulting in an extremely low density and unique elasticity.

**Figure 4:**
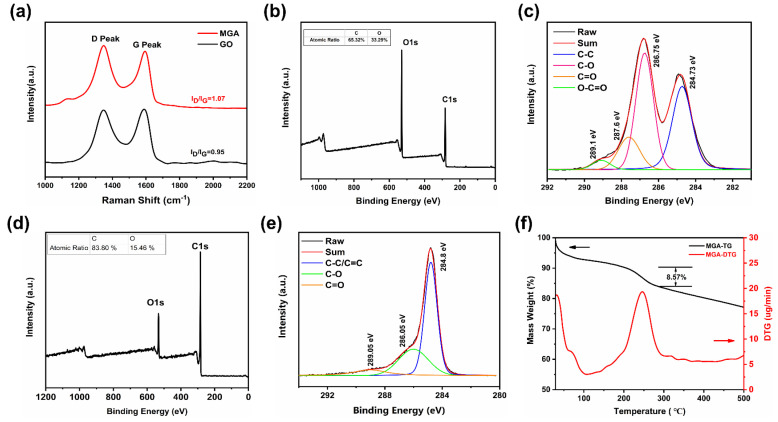
(**a**) Raman spectra of GO and MGA; (**b**) XPS spectra of GO; (**c**) C1sXPS spectra of GO; (**d**) XPS spectra of MGA; (**e**) C1sXPS spectra of MGA; (**f**) TG and DTG analysis of the MGA.

The laser Raman analysis of MGA and GO provided valuable insights into the structural changes that occur during hydrothermal reduction. The Raman spectrograms of GO and MGA show two different sets of characteristic peaks, the D-band at 1350 cm^−1^ (associated with the disordered region of graphene) and the G-band at 1590 cm^−1^ (associated with the graphitic region) [25,26], which were characteristic of carbon materials (Figure 4a). The I_D_/I_G_ ratio of GO was determined to be 0.95. for MGA, the I_D_/I_G_ ratio increased to 1.07. When graphene oxide was reduced, the defective regions were repaired, and the G-band of the Raman spectrum was enhanced, but there were more defects in it that were not repaired into the hexagonal ring structure, and these defective regions contributed more to the D-band of the Raman spectrograms, which resulted in that the I_D_/I_G_ of the reduced graphene oxide will be larger than that of the graphene oxide [27]. This result suggests that the hydrothermal reduction process led to the repair of the conjugated regions within the GO structure. During the hydrothermal treatment, a large number of carbon atoms in the sp^3^ hybridization mode were converted to sp^2^ hybridization, which will result in substantial off-domain π-bonds were formed between each carbon atom, and electrons could move freely within the off-domain π-bonds, which will restore the electrical conductivity of GO to some extent. The hydrothermal process led to the removal of oxygen-containing functional groups, repair of the graphene structure, and restoration of electrical conductivity, making MGA a promising material for various applications that require high electrical conductivity and a well-connected graphene framework.

XPS analysis was performed to further demonstrate the structural changes of rGO. The reduction efficiency of GO can be investigated by analyzing the XPS patterns, and related studies show that graphene oxide has a C/O of 1.96, while MGA has a C/O of 5.42 (Figure 4b–e), which indicates that most of the oxygen-containing functional groups in MGA have been removed, and the whole process had a high reduction efficiency. The C1s XPS pattern of MGA shows the peaks of three carbon atoms (Figure 4e), namely: non-carbon dioxide [28] (C-C/C=C, 284.8 eV), ether-bonded carbon [29] (C-O, 286.05 eV), and carbonyl carbon [30] (C=O, 289.05 eV). The C1s XPS patterns also illustrate that the carbon–oxygen correlation peaks are much weaker than those of GO in MGA, which indicates that GO was reduced to rGO.

TG analysis was used to study the pyrolysis process of MGA (Figure 4f). Some oxygen-containing functional groups remained inside the MGA after hydrothermal reduction, which has hydrophilicity, and the mass loss of MGA below 100 °C was mainly caused by the removal of internal bound water. Starting from 150 °C to the end of 300 °C, the TG curve showed a significant mass loss, and the mass of MGA decreased from 92.06% to 83.49% at this stage, with a loss value of 8.57%, which was due to the removal of unreduced oxygen-containing functional groups remaining in MGA [31]. The baseline of the DTG curve was higher above 300 °C than that around 100 °C, due to the internal MGA After 300 °C, the quality of MGA decreased slowly, which was related to the removal of the more stable oxygen-containing functional groups inside it. Most of the oxygen-containing functional groups inside MGA were removed in hydrothermal reduction, which was more favorable for the recovery of its electrical conductivity.

### 3.2. Mechanical Compression Performance Characterization of MGA-ETPU Sensors

**Figure 5:**
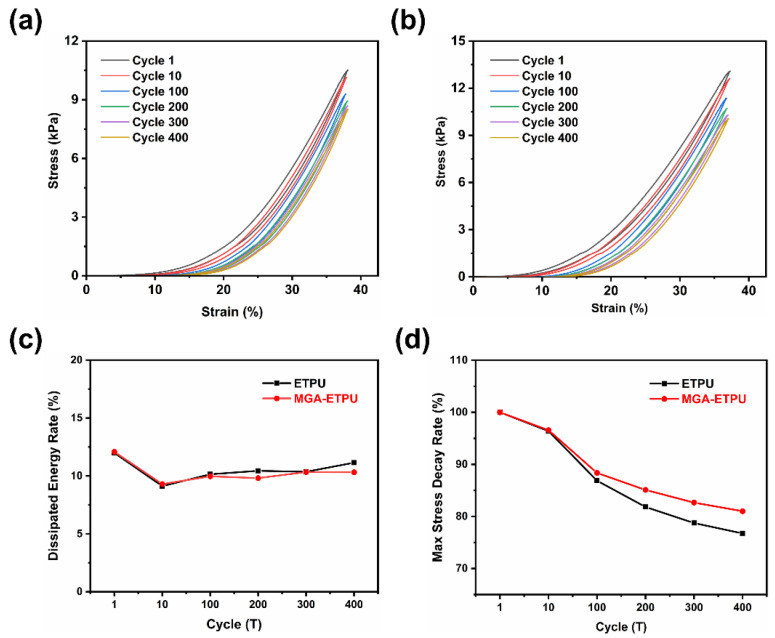
(**a**) MGA-ETPU with 400 load–unload cycles at 40% strain; (**b**) ETPU with 400 load–unload cycles at 40% strain; (**c**) dissipated Energy Rate of MGA-ETPU and ETPU in 400 load–unload cycles; (**d**) maximum Stress Decay Rate of MGA-ETPU and ETPU in 400 load–unload cycles.

Mechanical properties were another parameter to characterize the sensor performance. Four hundred load–unload cycles were performed at 40% strain on the MGA-ETPU sensor and a pure ETPU sheet of the same size. The stress–strain curves showed a narrow crescent shape (Figure 5a,b), and the maximum stress of the MGA-ETPU sensor decreased from 10.514 to 8.51 kPa during the whole cycle, a reduction of 19%, while the maximum stress of the pure ETPU sheet decreased from 13.085 to 10.61 kPa, a reduction of 18.9%. In addition, the stress–strain curves were integrated, and the amount of reduction in the integrated area was used to characterize the amount of loss in the performance of the material when subjected to the loading–unloading cycle test [32,33]. The maximum dissipated energy rate of MGA-ETPU was 11.14%, and the maximum stress decay rate was 23.25%. The maximum stress and performance loss decreased significantly between the first and the 100th cycle of the test for both groups of samples, and the amount of change stabilized after 100 cycles, due to the compression of the ETPU at the beginning of the compression cycle, when the internal molecules were squeezed and some of the molecular chain segments were brittle and fractured, and the performance loss gradually increased (Figure 5c,d), and the maximum stress that the samples could withstand decreased [28,34]. After 100 compression cycles, the internal structural changes of the samples stabilized, and therefore, the performance changes also stabilized, so the performance of the samples in the 100th cycle was less different from that in the 400th cycle. In addition, the narrow shape of the stress–strain curve of the sample indicates that the MGA-ETPU sensor has a low energy dissipation per unit volume and is almost an elastomeric material with high toughness and fatigue resistance [35], which can be used as a flexible sensor with excellent performance. The appearance of both groups of samples was not significantly deformed after loading and unloading cycles, indicating that the addition of MGA had little effect on the overall compression performance of the ETPU sheet after 400 loading and unloading cycles at 40% strain. After 400 loading–unloading cycle compression experiments, no significant deformation was found in the appearance of the two groups of samples, which indicates that ETPU, as a substrate for flexible sensors, can well resist compression deformation and can meet the needs of most flexible sensors for high strain performance. At the same time, comparing the experimental results of the two groups of samples, it can be found that the addition of MGA has little effect on the overall performance of ETPU, which is conducive to the long-term stable operation of MGA-ETPU sensors.

### 3.3. Pressure Sensing Performance of MGA-ETPU Sensor

**Figure 6:**
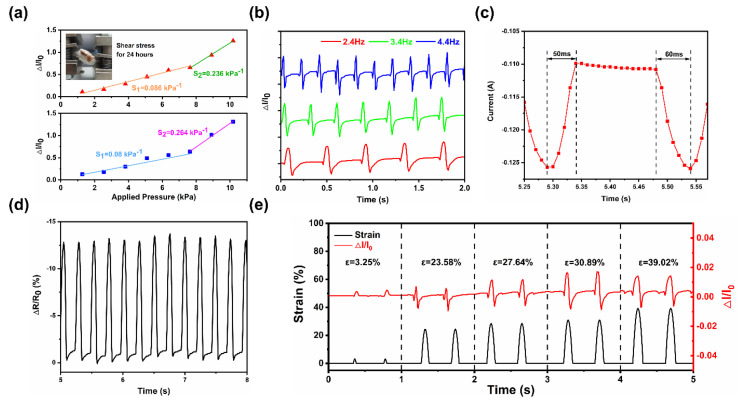
(**a**) Linear sensitivity in the range of 0.5 to 8 kPa, 8 to 10 kPa for MGA-ETPU; (**b**) dynamic response at a compression strain of 30.89% at frequencies of 2.4 to 4.4 Hz; (**c**) MGA-ETPU responds and recovers rapidly at 80% strain; (**d**) resistivity change curve of MGA-ETPU sensor under compression; (**e**) dynamic response of MGA-ETPU under different compressive strains.

After hydrothermal reduction of GO, the internal conjugate region was repaired, and the electrical conductivity was restored. The resistivity of MGA was measured to be 33.664 Ω·cm, and the conductivity was 0.024 S/cm, which has good electrical conductivity. We will conduct various sensing performance tests on the MGA-ETPU to verify its feasibility as a flexible piezoresistive sensor. Pressure from 0 to 10 kPa was applied to the MGA-ETPU and the current value of the sensor at each pressure was collected. The sensitivity of the MGA-ETPU was calculated by the formula *S* = (*∆I*/*I*_0_)/*∆P*, where *I*_0_ represents the current value of the sensor when no pressure is applied, *∆I* represents the change value of the current relative to *I*_0_ at a certain moment, and *∆P* represents the change value of the applied pressure [36]. The current response is shown in Figure 6a, from which it can be seen that there are two distinct linear regions of the sensor’s current accordingly, and the sensitivity of these two regions are 0.08 kPa^−1^ (0–8 kPa) and 0.264 kPa^−1^ (8–10 kPa), respectively. As the pressure applied to the MGA-ETPU increases, the internal compression of the MGA leads to an increase in the number of conductive pathways, which increases the strength of the response current and ultimately leads to an increase in sensitivity. The wide linear range of the MGA-ETPU while maintaining the sensitivity is a characteristic that makes it fully satisfy the requirements as a smart shoe sensor. Meanwhile, in order to verify the flexibility of MGA-ETPU, we applied shear stress to MGA-ETPU for up to 24 h. After this treatment, the appearance of MGA-ETPU was significantly deformed due to the presence of shear stress, and then we tested its electrical performance, which showed that after applying long-term shear stress, the sensitivity of MGA-ETPU did not change significantly in the low-pressure region (0–8 kPa) and decreased by 10.6% in the pressure region of 8–10 kPa. This is due to the fact that the long-term shear stress leads to permanent breakage of some covalent bonds in the carbon skeleton inside the MGA, which will increase the initial resistance of the MGA, decrease the electrical conductivity of the MGA, and ultimately reduce its sensitivity. However, after applying long-term shear stress, the MGA-ETPU still has excellent linearity, which proves its good flexibility, as shown in Figure 6a.

The flexible sensors are subjected to loads at different frequencies during use [37], and in order to test the dynamic response performance of the MGA-ETPU under loads at different frequencies, a strain of 30.89% was applied to the MGA-ETPU at 2.4, 3.4, and 4.4 Hz, respectively. As the frequency increases, the sensors exhibit similar current change curves (Figure 6b), which indicates that the sensors have better reproducibility when subjected to cyclic loads. The frequency test results indicate that the MGA-ETPU has high potential as a smart shoe flexible sensor. In addition to this, we analyzed the current response performance of the MGA-ETPU when subjected to compression cycling. At 80% compressive strain, the response time (50 ms) and recovery time (60 ms) of the MGA-ETPU to external pressure are very fast, which means that the sensor can quickly pick up rapidly changing signals (Figure 6c). The MGA-ETPU exhibits a significant resistance change when subjected to external pressure, which is favorable for signal processing. Excluding the error caused by the machinery, the two curves were almost symmetrical during loading and unloading, indicating that the resistance recovery rate of the MGA was almost as fast as the loading–unloading rate (Figure 6d). In order to evaluate the performance variation of the MGA-ETPU when subjected to different compressive strains, we tested the current response performance of the sensor under compressive strains of 3.25%, 23.58%, 27.64%, 30.87%, and 39.02%. The test results show (Figure 6e) that the current change of the sensor is gradually obvious with the increase of the applied compressive strain. The MGA-ETPU has a small distortion of the current change curve when subjected to a large compressive strain (*ε* = 39.02%), which is related to the structural damage of the internal MGA, but the sensor still has a more complete response period at this time. In particular, the sensor still has a more pronounced current change when a smaller compressive strain (*ε* = 3.25%) is applied, indicating that the sensor is still capable of recognizing weak signals.

**Figure 7:**
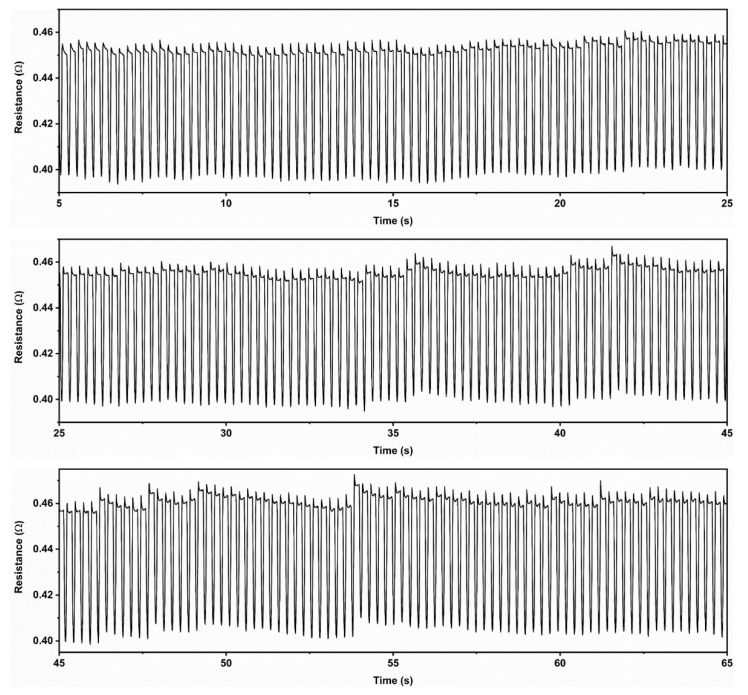
MGA-ETPU sensor resistance change over 300 compression cycles.

The MGA-ETPU was placed on a specialized rotary reciprocating device to perform axial extrusion experiments at 80% strain, and the change in current signal of the sensor was recorded on an electrochemical workstation (Figure 2c). Compression cycle stability experiments were conducted on the sensor by rapidly applying pressure to the sensor and then rapidly releasing it for 300 cycles within 60 s, and the resistance response is shown in Figure 7. The resistance of the sensor suddenly increased when it was compressed, and the resistance quickly returned to the initial position after releasing the pressure, and this characteristic would be maintained in each stage of the compression cycle. The results of the mechanical compression performance test showed that the MGA-ETPU sensor had excellent resilience. Therefore, these properties allow the MGA-ETPU sensor to be used in the field of flexible sensors.

### 3.4. Integrating MGA-ETPU into Smart Shoes

**Figure 8:**
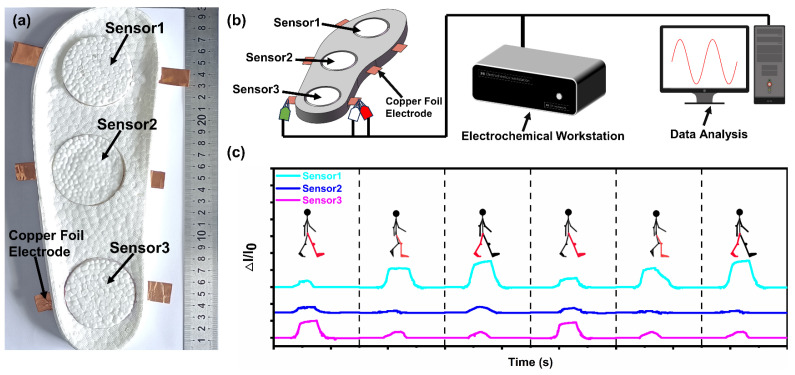
(**a**) Digital photograph of the sole with three MGA-ETPU sensors integrated; (**b**) smart shoe connected to an electrochemical workstation and data processing; (**c**) current change profiles of the three MGA-ETPU sensors in each walking state.

In order to verify the feasibility of using MGA-ETPU as a flexible sensor for smart shoes, an ETPU sole was fabricated using a hot-pressing process, and three MGA-ETPU sensors were integrated into the sole (Figure 8a). Electrochemical workstations were used to collect current data from sensors located in the front sole, center of foot, and heel when subjected to pressure (Figure 8b). For example, we used this smart shoe to test three typical states during a walking cycle, namely heel contact with the ground, sole contact with the ground, and forefoot contact with the ground. The three MGA-ETPU sensors have distinct signals in each state, and the sensor signals of the forefoot and heel are significantly stronger than those of the middle sensor (Figure 8c). By analyzing the current data of the relevant sensors, simple motion monitoring and gait recognition functions can be achieved.

## 4. Conclusions

In summary, MGA was prepared using the modified Hummers’ method and the Pickering emulsion template method with hexane as the oil phase, in which n-hexane acted as a soft template, and its properties were tested. Our MGA had a very low apparent density (3.02 mg/cm^3^) and a large number of closed-pore structures with pore diameters of about 100 μm. The pore wall was a three-dimensional wrinkled structure formed by stacking a large number of rGO lamellae, which allowed the pore wall to withstand a large amount of deformation, and the deformed pore wall returned to the initial state after withdrawing the external force. The MGA-ETPU sensor, assembled using MGA and ETPU as the substrate, demonstrated excellent elasticity and low energy loss per unit volume. After 400 loading and unloading cycles at 40% strain, the maximum stress of the sensor was reduced by 19%, and the amount of energy loss per unit volume stabilized after 100 cycles. The hydrothermal reduction method repaired a part of the conjugate region of GO, which restored the electrical conductivity (the resistivity of MGA was 33.664 Ω·cm, and the electrical conductivity was 0.024 S/cm). The MGA-ETPU has a large linear sensing range (0 to 10 kPa) and consists of two linear regions: the low-pressure region (0 to 8 kPa) and the high-pressure region (8 to 10 kPa), with sensitivities of 0.08 and 0.246 kPa^−1^, respectively. The electrical performance test results showed that when the sensor was compressed, the resistance change rate was 12.5% and returned to the original state within 0.05 s. Overall, the study demonstrated that MGA possesses not only good electrical conductivity but also excellent elasticity, making it a suitable material for flexible mechanical sensors. The MGA-ETPU sensors, with their remarkable electrical conductivity, sensitivity, and flexibility, can find applications in various fields, such as shoes, shock-absorbing materials, cushioning materials, and flexible sensors. These findings provide a solid foundation for further research and development in this area.

## Data Availability

Not applicable.
